# An analysis of the regulatory program of quality audits in radiotherapy in Brazil from 1995 to 2007

**DOI:** 10.1120/jacmp.v12i2.3330

**Published:** 2011-01-30

**Authors:** Eduardo de Paiva, Luiz A. R. da Rosa, Ricardo R. A. Brito, Lidia V. de Sá, Ana C. M. Dovales, Delano V. S. Batista, Ricardo A. Giannoni, Alexandre F. Velasco

**Affiliations:** ^1^ Instituto de Radioproteção e Dosimetria – IRD/CNEN/MCT Rio de Janeiro Brazil

**Keywords:** radiotherapy, audit, quality assurance, dosimetry, radiation protection

## Abstract

The Brazilian Institute of Radiation Protection and Dosimetry (IRD/CNEN) carried out quality assurance regulatory audits in Brazilian radiotherapy facilities from 1995 to 2007. In this work, the set of data collected from 195 radiotherapy facilities that use high‐energy photon beams are analyzed. They include results from audits in linear electron accelerators and/or Co‐60 units. The inspectors of IRD/CNEN performed the dosimetry of high‐energy radiotherapy photon beams according to the IAEA dosimetry protocols TRS 277 and TRS 398, and the values of measurements were compared to stated values. Other aspects of radiological protection were checked during on‐site audits such as calibration certification of clinical dosimeters and portable monitors, existence and use of check source, use of barometer and thermometer, individual dose registry and training of staff. It was verified that no check source was available in 38% of the visited facilities; the training of personnel was not adequate in 9% of the facilities and the registry of accumulated individual doses was not being done in 6% of the facilities. Measurements of absorbed dose have indicated deviations in the range ± 3% for 67.6% of the cobalt‐60 units and 79.6% of medical linear accelerators; 18.5% of Co‐60 irradiators and 9.6% of linear accelerators presented deviations in the range 3% <δ≤ 5%. Finally, 13.9% of Co‐60 facilities and 10.8% of linear accelerator facilities presented dosimetry deviations above 5%. The effort in dosimetric quality control performed by IRD/CNEN audits has yielded positive changes that make radiation treatment facilities more reliable.

PACS number: 87.55.Qr

## I. INTRODUCTION

Quality assurance in radiotherapy is broad in scope, encompassing all activities needed to ensure the safe, accurate and consistent implementation of the physician's prescription. As such, quality assurance in radiotherapy should involve all members of the clinical team, with a collective focus on the interdependence of the many steps in the process.

One very important part of overall quality assurance is quality control. Quality control is the regulatory process used to measure actual quality performance, based on existing national standards and international recommendations, and the actions necessary to keep or to recuperate conformance with those standards and recommendations.

The quality control process should be carried out not only by the radiotherapy facility staff, at a very important fundamental stage, but also by the regulatory authority. The regulatory authority executes that task through quality audits. A quality audit is a systematic and independent examination to determine whether quality activities and results comply with planned arrangements, and whether the arrangements are effectively implemented and suitable to achieve the stated objectives. Quality audits should be performed by personnel not directly responsible for the areas being audited, preferably in cooperation with the incumbent staff.

Practical quality audits can be performed by two ways: using mailed dosimeters (the so‐called postal audit), or through quality audit visits. Postal audits use, usually, thermoluminescent dosimeters (TLD) and can include procedural audits by using a questionnaire.^(^ ^1^ ^,^ ^2^ ^)^ Quality audit visits can evaluate practical aspects in detail, limited only by time. They can audit procedural aspects by questioning staff and by inspection of procedures and records. Additionally measurements can be performed and then compared to the records presented by the radiotherapy facility staff.^(^ ^3^ ^,^ ^4^ ^)^

The audit programs at radiotherapy facilities and the use of postal systems for quality control of the therapeutic doses are not anything new. The pioneers in this area were the Radiological Physics Center (RPC) of the M. D. Anderson Cancer Center, University of Texas, USA since 1968,^(5)^ and the International Atomic Energy Agency (IAEA) which, since 1969, has been conducting a program of postal dose evaluation in radiotherapy using thermoluminescent detectors.^(1)^

The content of a quality audit visit should be predefined and will depend on the purpose of the visit. For example, is it a routine regular visit within a national or regional quality audit network, or is it a visit following an observed higher‐than‐expected deviation in a mailed TLD audit program that the radiotherapy facility cannot explain?

The following is an example of the contents of a quality audit visit:

- Check documentation: documents on policies and procedures, quality assurance program structure and management, patient dosimetry procedures and treatment delivery procedures.
- Check infrastructure: equipment, personnel and radiation protection program.
- Carry out check measurements of beam calibration and environmental doses inside and outside the radiotherapy facility, survey monitoring.

In Brazil, the national regulatory body in charge of the licensing and control of nuclear and radioactive practices and installations, according to national standards^(^ ^6^ ^–^ ^10^ ^)^ and following international recommendations,^(^ ^11^ ^–^ ^14^ ^)^ is the National Commission of Nuclear Energy (CNEN). In the CNEN organizational structure there is a division responsible for inspecting and regularly evaluating the licensed installations, with different groups acting in industry and medicine fields. In this context, there is a trained group dedicated to carry out radiotherapy facility quality audit visits. Until 2007, this group was linked to CNEN's Institute of Radiation Protection and Dosimetry (IRD/CNEN). The group produced quality audit visit reports to the General Coordination of Medicine and Industry of CNEN (CGMI), which is responsible for the evaluation of the licensed radiotherapy installations. Since 2008, this group belongs to CGMI. IRD/CNEN quality audit visits are carried out every two years.

During IRD/CNEN quality audit visits, the following items are considered:

- **Documentation inspection** that embodies:
  - Quality assurance program structure and management documentation
  - Quality control data of equipments and procedures, including certified updated calibration factors
  - Dosimetry procedures documentation and dosimetric data reports
  - Radiation protection data records, including facility dose monitoring survey reports and individual dose reports, and actions implemented (when necessary)
  - Staff training documentation including the topics taught and the attendance frequency to the lectures and practices signed by the trainees
- **Radiotherapy facility dose monitoring survey**, including a check of the adequacy of radiological protection barriers. It is important to mention that only photon ambient dose values are considered for the monitoring survey, although IRD/CNEN recognizes and has been investigating the importance of the photoneutron contribution for the ambient doses in radiotherapy facilities possessing linear accelerators (LINACs) presenting X‐ray therapeutic beams with maximum energies higher than 10 MeV.^(^ ^15^ ^,^ ^16^ ^)^
- **Dosimetry of the therapeutic beams**, which consists of the following steps:
  - The medical physicist is requested to set up experimental conditions in such a way that a specific radiation field should be delivered to an ion chamber at reference conditions.^(3)^
  - The medical physicist is also requested to calculate the time or monitor units necessary to deliver an absorbed dose‐to‐water value of 100 cGy to the geometric center of the sensitive volume of the ion chamber.
  - The measured absorbed dose is then compared to the requested values.

Presently there are 195 radiotherapy centers in Brazil operating 160 clinical linear accelerators and 86 therapeutic cobalt‐60 units. These numbers are being constantly modified, with the decommissioning of older treatment equipments that are replaced by one or more modern therapeutic irradiators and the beginning of operation of new radiotherapy facilities. Brazil is divided into five geographic regions. The distribution of radiotherapy facilities in these regions is 60% in the southeast region, 20% in the south region, 15% in the northeast region, 4% in the mid west region and 1% in the north region.

This work presents the quality audit data obtained by the inspectors of IRD/CNEN during visits to Brazilian radiotherapy facilities from 1995 up to 2007. An analysis is developed in terms of annual noncompliances occurrences in order to identify a possible improvement of the performance of radiotherapy facilities as a response to the quality control audit IRD/CNEN program.

## II. MATERIALS AND METHODS

By checking the documentation presented by the person responsible for the radiotherapy facility, in the period 1995–2007 IRD/CNEN staff could verify different aspects of radiological protection, including those for workers, patients and the public in general. Additional insight into the quality assurance and radiation safety programs in place at the facility could also be provided. Test of congruence between radiation field and light field was also an item reviewed during the quality audit inspections.

During the radiometric survey, measured ambient dose rates, ${\dot{H}}^{*}$, are compared to derived limits for controlled and uncontrolled areas, according to the classification of the area under consideration. In Brazil, these limits are 0.4 and 0.02 mSv/week, respectively, for controlled and uncontrolled areas,(7) although the publication NCRP 151(17) suggests the value of 0.1 mSv/week for controlled areas.

Radiometric survey was carried out placing the survey monitors at a distance of 30 cm from the point of interest on the surface of the radiological protection barrier. The equipments used by IRD/CNEN inspectors were ion chamber type survey meters SmartION models 2120 R, SN 4628 and SN 4779 (Thermo Fisher Scientific, Wiltham, MA), and Inovision models 451P, SN541 and SN542 (Inovision Software Solutions Inc., Chesterfield, MI). The equipments are calibrated every year in the Brazilian Secondary Standard Dosimetry Laboratory (SSDL) in Rio de Janeiro. The uncertainty associated to calibration factors is better than 10% for a confidence level of 95%.

The inspectors of IRD/CNEN have carried out the dosimetry of high‐energy photon radiotherapy beams according to the International Atomic Energy Agency (IAEA) dosimetry protocols TRS 277^(18)^ and TRS 398.^(19)^ The clinical dosimeters used consisted of electrometers Standard Imaging model MAX 4000 (Standard Imaging, Middleton, WI), Nuclear Enterprises model 2570/1B (Nuclear Enterprises, Wichita, KS), and PTW models 10001 and 10002 (PTW, Freiburg, Germany), as well as ion chambers PTW model TN30013 and Nuclear Enterprises models 2571 and 2571A. The dosimetric systems are calibrated at intervals of two years in the Brazilian Secondary Standard Dosimetry Laboratory (SSDL) in Rio de Janeiro. The standard uncertainty in clinical dosimeter factor is lower than 1.5% for a confidence level of 95%.

Quality control audit *in loco* dosimetric measurements are described by da Rosa et al.^(3)^ The radiotherapy facility medical physicist is requested to calculate the time (in the case of cobalt‐60 irradiators) or the number of monitor units (in the case of electron linear accelerators) necessary to deliver an absorbed dose value of 100 cGy in the sensitive volume of the ion chamber.

The percent dosimetric deviation, δ, between the requested absorbed dose, $D_{R}$, and the mean measured dose, $D_{M}$, is determined according to the formula:

(1) $$\delta =\frac{D_{M}-D_{R}}{D_{R}}\times 100$$

An acceptable percent deviation should not be out of the range −3% to +3%. This criterion was adopted taking into account that in radiotherapy, considering all sources of uncertainties, the total deviation between the prescribed and administrated doses should be in the range ± 5%.^(20)^ These uncertainties consist basically of dosimetric (dosimeter calibration, dose calculation, reference point of measurement) and geometric uncertainties (setup, organ motion, patient position).

Considering all sources of uncertainties involved in high‐energy photon beam dosimetry process, including those related to pressure and temperature measurements for the clinical dosimeter response correction, dosimetry results are given with a total uncertainty better than ± 3% for a confidence level of 95%.

## III. RESULTS

About thirty percent of the radiotherapy facilities existing in Brazil operate without authorization from the regulatory authority because the authorizations expire and, due to different reasons, they are not renewed; as well, there are always new facilities requiring an authorization to operate.

During the inspection, much information about the radiotherapy facility staff was collected. Of particular note is the inadequate number of radiation safety officers and radiation oncologists. Considering the acceptable situation of at least one radiation safety officer and one radiation oncologist per radiotherapy facility, there is a total deficit of 25% and 37%, respectively, for those professionals. An immediate consequence of this situation is that there are professionals working in more than one radiotherapy facility, meaning that they do not work full‐time in the facility.

**Figure 1:**
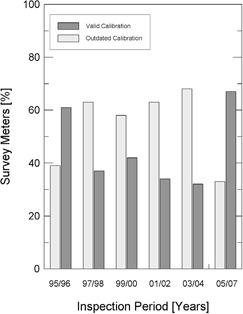
Percentage of survey monitors with valid calibration factors and with outdated calibration factors, according to the inspection period considered.

**Figure 2:**
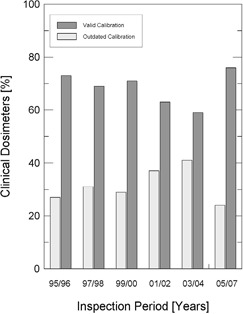
Percentage of clinical dosimeters with valid calibration factors and with outdated calibration factors, according to the inspection period considered.

Another item taken into account was the validity of the calibration factor of survey monitors and clinical dosimeters employed by the radiation safety officers and the medical physics staff working in the radiotherapy facility. Figures 1 and 2 present the existing situation of valid calibration equipments and outdated calibration equipments, for each inspection period considered, respectively, for survey monitors and clinical dosimeters.

The existence of check sources for monitor and dosimeter routine tests was also checked. In 38% of the radiotherapy facilities inspected, it was observed that there was no test source available for routine performance tests of survey monitors and clinical dosimeters. This noncompliance can be one of the causes of some other noncompliances observed during survey monitoring audits and clinical beam dosimetry audits.

The radiological protection plan approved by CNEN is the document that formalizes the actions that should be taken by the radiation protection officer of the radiotherapy facility when necessary. Therefore, that plan is always checked during on‐site visits to radiotherapy facilities. In 21% of the facilities audited, the radiological protection plan document was unavailable. Either there was no radiological protection training plan for radiotherapy professionals or the training plan was not adequate or was applied improperly in 9% of the radiotherapy facilities checked. The quality control of equipment was not performed or was not registered in 11% of the facilities visited. The record of individual dose accumulated by the workers was not being carried out in 6% of the radiotherapy facilities.

The coincidence between radiation fields and light fields is another item reviewed during the quality audit inspections. In general, noncompliances relating to this item are very rare, considering that the coincidence should occur within the limit of 2 mm. Results indicated that about 99% of these tests satisfied the criterion of a 2 mm congruence.

Survey monitoring audits indicated that the most critical noncompliances were observed in uncontrolled areas in the vicinity of ${}^{60}\text{Co}$ facility rooms where measured dose rates values like 0.55 and 4.5 mSv/week, (as reported^(21)^), were obtained. In many cases the facilities were subjected to nonauthorized project changes, or the person responsible for the facility decided to utilize a higher activity ${}^{60}\text{Co}$ source not appropriate to the existing approved irradiator bunker shielding^.(^ ^21^ ^)^ Another possible reason is a ${}^{60}\text{Co}$ unit was positioned at an unusual configuration (for instance, forming angles about 45° with the bunker walls), a situation that can generate higher dose rates in the neighborhood of the treatment room.^(22)^

Although the Brazilian legislation does not stipulate a maximum deviation for the absorbed dose value at the reference depth (5 or 10 cm in water according to the quality of the clinical radiation beam), IRD/CNEN adopted for the purpose of the national program of quality audits in radiotherapy facilities the criterion of 3% as the absolute maximum acceptable agreement between the dose measured by IRD/CNEN staff and the dose indicated by the radiotherapy facility staff. IRD/CNEN does not recommend or require a specific calibration protocol. By the year 1999, there were few facilities that still used the IAEA protocol TRS 110.^(23)^ The IAEA protocol TRS 277^(18)^ was used by all radiotherapy facilities from 2000 to 2005. Since 2005, many radiotherapy facilities have been using the IAEA protocol TRS 398.^(19)^ At the end of 2007, the IAEA protocol TRS 398 was used by 70% of facilities and the IAEA protocol TRS 277 was used by the remaining 30% of facilities.

**Figure 3:**
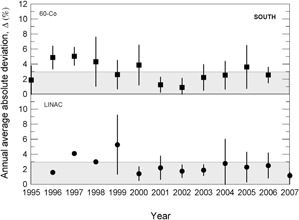
Annual average absolute percent dosimetric deviation values, Δ, for Brazilian south region. Error bars are one standard deviation of the mean absolute value.

**Figure 4:**
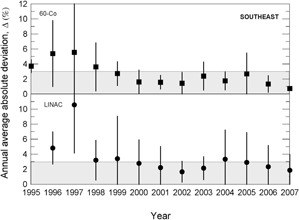
Annual average absolute percent dosimetric deviation values, Δ, for Brazilian southeast region. Error bars are one standard deviation of the mean absolute value.

**Figure 5:**
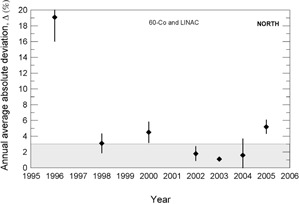
Annual average absolute percent dosimetric deviation values, Δ, for Brazilian north region. Error bars are one standard deviation of the mean absolute value.

**Figure 6:**
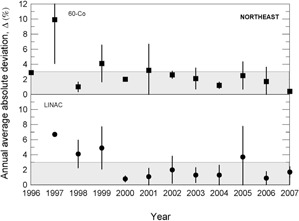
Annual average absolute percent dosimetric deviation values, Δ, for Brazilian northeast region. Error bars are one standard deviation of the mean absolute value.

**Figure 7:**
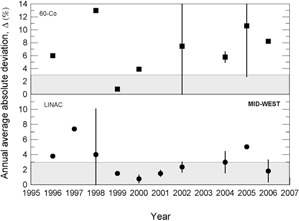
Annual average absolute percent dosimetric deviation values, Δ, for Brazilian mid‐west region. Error bars are one standard deviation of the mean absolute value.

During the 13 years of data collection for this work, about 800 clinical beam dosimetry measurements in linear accelerators with accelerating voltages in the range 4– 15 MV, and in cobalt‐60 irradiators were carried out. The percent dosimetric deviation, δ, between the requested absorbed dose, $D_{R}$, and the mean measured dose, $D_{M}$, is determined according to Eq. (1). Considering the large territory of the country, the data were grouped by Brazilian official geographic regions in terms of annual average absolute deviation values, Δ, as presented in Figs. 3 to 7, and absolute deviation values, δ, as shown in Table 1.

**Table 1:** Overview of δ values evaluated during audits in radiotherapy facilities in Brazil. In parentheses it is shown the total number of audits carried out in relation to the number of treatment equipment available in the region and also to the time that they are available.

*Absolute Deviation (%)*	*South (% of audits)*		*Southeast (% of audits)*		*North (% of audits)*		*Northeast (% of audits)*		*West (% of audits)*		*Total (%)*	
	*Co*	*LINAC*	*Co*	*LINAC*	*Co*	*LINAC*	*Co*	*LINAC*	*Co*	*LINAC*	*Co*	*LINAC*
δ≤3	63 (41)	83 (77)	74 (122)	78 (202)	25 (3)	85 (6)	70 (38)	82 (65)	42 (5)	76 (32)	67.6	79.6
3<δ≤5	22 (14)	12 (11)	18 (29)	10 (25)	25 (3)	15 (1)	17 (9)	5 (4)	16 (2)	12 (5)	18.5	9.6
δ>5	15 (10)	5 (5)	8 (15)	12 (32)	50 (6)	0 (0)	13 (7)	13 (10)	42 (5)	12 (5)	13.9	10.8

The results presented in Figs. 3 to 7 show, in general, a decrease of δ and Δ values with an increase in the numbers of audit inspections to which the radiotherapy facility was subjected. This behavior can indicate a positive response of radiotherapy facilities to IRD/CNEN inspections in the sense that they are giving more attention to dosimetry and quality control procedures. In Fig. 5, which presents the data for the Brazilian north region, the same symbols were used for LINACs and Co‐60 irradiators, due to the low statistics.

Table 1 summarizes the values of δ measured in Brazil during 13 years of audits The results for most of Co‐60 units and linear accelerators audited are in the range ± 3%, respectively 67.6% and 79.6%; 18.5% of the audits in Co‐60 facilities and 9.6% of the audits in linear accelerator facilities presented deviations in the therapeutic beam dosimetry in the range 3% <δ≤ 5%. Deviations in therapeutic beam dosimetry higher than 5% were measured, respectively, in 13.9% and 10.8%, of Co‐60 irradiators and linear accelerators audits. The ICRU Report 24 recommends a maximum deviation of 5%.(20) It should be noted that, in general, δ values for Co‐60 irradiators are worse than those for linear accelerators. This result can be explained on the basis that linear accelerators are more complex equipments to which more rigorous and detailed quality assurance programs are applied.

**Figure 8:**
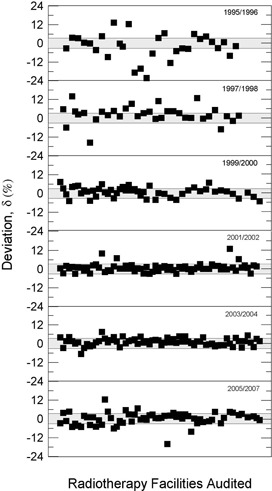
Percent dosimetric deviation, δ, between the requested absorbed dose, $D_{R}$, and the mean measured dose, $D_{M}$, for cobalt‐60 irradiators, for all Brazilian regions, from 1995 to 2007.

**Figure 9:**
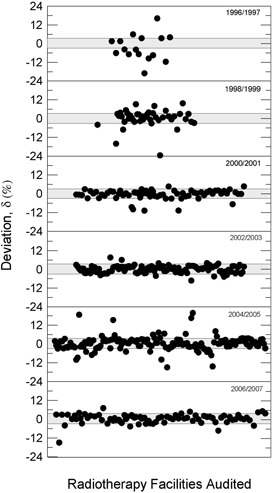
Percent dosimetric deviation, δ, between the requested absorbed dose, $D_{R}$, and the mean measured dose, $D_{M}$, for linear accelerators, for all Brazilian regions, from 1996 to 2007.

Figures 8 and 9 show that, for cobalt‐60 irradiators (squares) and linear accelerators (circles), respectively, the percent dosimetric deviation, δ, between the requested absorbed dose, $D_{R}$, and the mean measured dose, $D_{M}$, for the whole country from 1995 up to 2007. It can be noted that most of the deviations are in the range ± 3%, although some results were, in modulus, higher than 3%. For instance, in 1999 a negative deviation of −23.4% for a 6 MV linear accelerator was measured. A positive deviation of 19.7% for a 15 MV linear accelerator was measured in 2005. A δ value equal to −15% was measured for a 6 MV linear accelerator in 2006. In 1996 δ values varying from −22.3% to −12.5% for different telecobalt units were found and, in 2005, a δ of −16.2% was determined for a unit of cobalt‐60. In Table 2, the worst δ values (absolute values greater than 10%) and the main possible sources of errors are summarized.

**Table 2:** The worst δ values and the main possible sources of errors encountered during regulatory audits in therapeutic beam dosimetry from 1995 to 2007.

*Case No.*	*Equipment*	*Year*	*Percent Deviation*	*Sources of Errors*
1	Co‐60	1996	13.4	No check source used
2	Co‐60	1996	−12.5	No check source used
3	Co‐60	1996	12.4	No barometer used
4	Co‐60	1996	−22.3	Misuse of dosimetry protocol
5	Co‐60	1996	−18.7	Misuse of dosimetry protocol
6	Co‐60	1996	−16.2	Misuse of dosimetry protocol
7	LINAC 6 MV	1997	−19.0	Physicist not properly trained
8	LINAC 6 MV	1997	16.3	Wrong factors applied to dose calculation
9	LINAC 6 MV	1997	−11.6	Physicist not properly trained
10	Co‐60	1997	−15.5	Wrong factors applied to dose calculation
11	Co‐60	1997	14.0	Uncalibrated dosimeter
12	Co‐60	1998	11.0	No check source used
13	LINAC 6 MV	1998	−16.0	Uncalibrated dosimeter
14	Co‐60	1998	13.0	Uncalibrated dosimeter
15	LINAC 6 MV	1999	−23.4	Wrong factors applied to dose calculation
16	LINAC 4 MV	2001	−10.6	No check source used
17	LINAC 4 MV	2001	−10.7	No check source used
18	Co‐60	2001	10.0	Physicist not properly trained
19	Co‐60	2002	13.0	Uncalibrated dosimeter
20	LINAC 4 MV	2004	15.2	Physicist not properly trained
21	LINAC 10 MV	2004	−10.2	Wrong factors applied to dose calculation
22	LINAC 6 MV	2004	18.6	Wrong factors applied to dose calculation
23	LINAC 6 MV	2005	−15.0	Uncalibrated dosimeter
24	LINAC 6 MV	2005	−10.1	Physicist not properly trained
25	Co‐60	2005	−16.2	Wrong factors applied to dose calculation
26	Co‐60	2005	12.3	No check source used
27	LINAC 10 MV	2005	16.4	No check source used
28	LINAC 15 MV	2005	19.7	No check source used
29	LINAC 6 MV	2005	−14.4	Uncalibrated dosimeter
30	LINAC 6 MV	2006	−15.0	Uncalibrated dosimeter

The importance of IRD/CNEN biennial inspections for the reduction of δ deviations can be observed from data in Figs. 3 to 7, where annual average absolute deviation values, Δ, are presented together with correspondent standard deviations. It can be noted that, in general, Δ values decrease with the biennial continuous visits of IRD/CNEN inspectors to the facilities.

**Figure 10:**
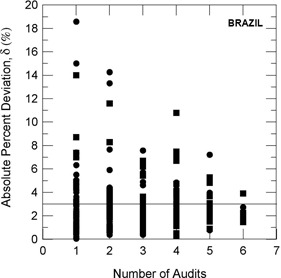
Relationship between the last absolute value of δ measured for each therapeutic irradiator and the number of audits already completed by these irradiators. Linear accelerators are represented by circles and Co‐60 irradiators by squares.

Figure 10 depicts the last absolute value of δ measured for each therapeutic irradiator with the number of audits already completed by these irradiators. It is easily observed that δ values decrease with the increase of the number of audits, indicating the important role that the IRD/CNEN biennial program of audits plays to enhance the effectiveness of cancer treatment.

## IV. DISCUSSION

The Brazilian regulatory authority should consider with more emphasis the existence of radiation safety officers and radiation oncologists working in more than one radiotherapy facility, mainly when the radiotherapy facilities are very far from each other, sometimes localized in different states of the country. The Brazilian regulatory authority should verify the ability of these professional to be physically available on site within a reasonable time in case of urgent need.

Even considering the large quantity of radiotherapy facilities in Brazil and the size of the country, it is interesting to observe homogeneity in the use of dosimetry protocols by the facilities. Until 2005, the majority of the radiotherapy facilities used the IAEA protocol 277.^(18)^ In 2007 many radiotherapy facilities started using the IAEA protocol 398.^(19)^ This decision permitted the uniformity and the dissemination of dosimetric procedures all over the country.

In the work of dosimetry quality audits of high‐energy photon beams in Greece,^(4)^ was reported that some facilities did not use a dosimetry protocol in order to conduct the regular therapeutic beam dosimetry of cobalt‐60 units. They substituted the dosimetric measurements by the correction of the irradiator output, considering only the source decay. In the present study, no ${}^{60}\text{Co}$ facility investigated substituted the regular therapeutic beam dosimetry using a dosimetry protocol by the correction of the irradiator output considering the source decay.

Figures 8 and 9 show the absorbed dose percentage deviations evaluated from 1995 to 2007 for telecobalt irradiators and clinic linear accelerators. Several noncompliances, deviations out of the range ± 3%, can be observed – mainly for units of ${}^{60}\text{Co}$. Many of these facilities were obsolete and were subsequently replaced or modernized.

The largest deviations shown in Figs. 8 and 9 and their possible causes are listed in Table 2. The worst result found was −23.4% (Fig. 9) for a 6 MV Linac in 1999. A investigation team of inspectors from the IRD/CNEN discovered that the professional responsible for the beam dosimetry had applied wrong factors in the calculation of monitor units. A deviation of −22.3% (Fig. 8) was found in 1996 for a ${}^{60}\text{Co}$ unit as a result of misuse of the dosimetry protocol. In 2005 a deviation of 19.7% (Fig. 9) was observed for a 15 MV Linac, possibly due to the absence of clinical dosimeter quality control procedures. The facility did not have, or did not use, any source test. In 1998 a linear accelerator dosimetry audit produced a deviation of −16% (Fig. 9). The facility had no clinical dosimeter calibrated. Many medical physicists and radiation protection officers claim that it is very difficult to schedule the equipment calibration, clinical dosimeter or survey monitor, in a Brazilian‐certified calibration laboratory.

Other causes relating to the large deviations listed in Table 2 are the inadequate training of some radiotherapy facility staffs and the lack of barometers in some facilities. In many installations, the value of the atmospheric pressure is informed by airports, which are not always close to the radiotherapy facility and not always at the same altitude of the facility. Figures 3 to 7 show the annual absolute mean percent deviations over the period studied, Δ, and respective standard deviations. It can be seen that, in general, the deviations decrease with time, indicating a positive response of radiotherapy facilities to IRD/CNEN audits. The existence of this positive response to IRD/CNEN audits is reinforced by Fig. 10, in which it can be observed a clear trend to smaller dosimetry deviations associated with more audited facilities.

## V. CONCLUSIONS

IRD/CNEN audits in radiotherapy facilities were very effective during the 13 years analyzed in this investigation, helping to identify different kinds of nocompliances and contributing to more accurate and consistent radiotherapy in Brazil.

The Brazilian regulatory authority should put an additional effort into the regularization of the licenses of operation that are not current, and special attention should be paid to the update of clinical dosimeters and survey monitors calibration certificates. However, it is important to point out that the latest results, for the years 2005–2007, already indicate a higher proportion of equipment with valid calibration certificates.

Considering the radiological protection of the staff working in radiotherapy facilities, as well as that of the public in general, more attention is needed to facilities that have Co‐60 irradiators, since the dose rates measured in controlled and uncontrolled areas are sometimes near to or higher than the dose limits required by norms for them. These inadequate values are normally detected after the substitution of a Co‐60 decayed source.

The results of this study indicate that quality assurance audits are an effective tool to help ensure accurate and safe operation of radiotherapy facilities.
